# Delineation of two intracranial areas and the perpendicular intracranial width is sufficient for intracranial volume estimation

**DOI:** 10.1007/s13244-017-0583-0

**Published:** 2018-01-26

**Authors:** Niklas Klasson, Erik Olsson, Carl Eckerström, Helge Malmgren, Anders Wallin

**Affiliations:** 1 Institute of Psychiatry and Neurochemistry, Department of Neuroscience and Physiology, The Sahlgrenska Academy, University of Gothenburg, Wallinsgatan 6, Box 400, 405 30 Gothenburg, Sweden; 2 Institute of Medicine, Department of Internal Medicine and Clinical Nutrition, The Sahlgrenska Academy, University of Gothenburg, Box 400, 405 30 Gothenburg, Sweden

**Keywords:** Estimation, Intracranial volume, Intracranial area, Magnetic resonance imaging, Manual segmentation

## Abstract

**Objectives:**

The aim of the present study is to determine if the delineation of one or two optimally chosen intracranial areas (ICA) is enough to achieve adequate estimates of intracranial volume (ICV) in magnetic resonance imaging.

**Methods:**

The correlations of 62 fully delineated ICVs with four types of ICV estimates were calculated. The estimate types were: (1) a single midsagittal ICA, (2) single ICA multiplied by the intracranial width perpendicular to the ICA, (3) sum of two ICAs multiplied by the perpendicular intracranial width and (4) shape-preserving piecewise cubic interpolation using two ICAs. For methods 2–4, the fully delineated ICVs were randomly separated into an evaluation and a validation set of equal size. Method 1 was validated against all of the fully delineated ICVs.

**Results:**

Estimates from method 1 had a Pearson correlation of 0.904 with fully delineated ICV. For method 2, the correlation was 0.986 when delineating the sagittal ICA at 31% of the sagittal intracranial width. For methods 3 and 4, the correlations were both 0.997 when delineating the sagittal ICAs at 17.5 and 64% and at 12 and 64% respectively.

**Conclusions:**

Delineation of two specific intracranial areas is sufficient for intracranial volume estimation.

***Main messages*:**

*• Delineation of two specific intracranial areas is sufficient for intracranial volume estimation.*

*• The estimates had a Pearson correlation of 0.997 with intracranial volume.*

*• The estimation should take no more than 5 min.*

**Electronic supplementary material:**

The online version of this article (10.1007/s13244-017-0583-0) contains supplementary material, which is available to authorized users.

## Introduction

Estimates of intracranial volume (ICV) in magnetic resonance (MR) imaging are mainly used to reduce variance in regional brain volumes of interest. To reduce variance caused by differences in ICV, linear regression has been suggested [1–3]. Then, the variance reduction is limited by the squared Pearson correlation of the ICV estimate with ICV. In a study by Voevodskaya et al., ICV estimated by FreeSurfer explained about 16% of the variance in hippocampal volume of healthy elderly [2]. A variance reduction from 16 to 1 % through normalisation would require an ICV estimate with a Pearson correlation with ICV of 0.97 ([1–0.01/0.16]^0.5^).^1^

Manual delineation of the inner table of the whole skull is presumably the most valid method for estimation of ICV in MR images, but takes hours [4]. It is instead common to use estimates calculated from every tenth intracranial area (ICA) [5–8]. The time needed for such estimates is about 15 min [4], and the estimates have been shown to be highly valid for MR acquisitions with around 1 mm^3^ voxels [4, 9]. By increasing the spacing of the ICAs, the time needed for the estimation drops, but so does the validity [4, 9]. Delineating two to three ICAs with 50-mm spacing can result in correlations around 0.96–0.99 and takes less than 5 min [4], while using one mid-sagittal ICA lowers the correlation to about 0.88–0.89 [10, 11].

Possibly, one or two ICAs delineated at optimal positions is enough to achieve correlations of 0.99 with fully delineated ICV, since this figure is within the upper range of the correlations found when delineating ICAs with 50 mm spacing. The intracranial width perpendicular to the ICAs could serve to determine the positions of the ICAs and to increase the validity of the estimates.

The aim of the present study is to determine if the delineation of one or two optimally chosen intracranial areas (ICA) is enough to achieve adequate estimates of ICV.

## Materials and methods

### Participants

All participants in the present study are part of the Gothenburg MCI (mild cognitive impairment) study [12, 13]. The participants are either patients referred to a memory clinic or controls recruited from organisations for seniors. The Gothenburg MCI study has been ethically approved (diary no. L091–99, 1999, T479–11, 2011), and all participants have given their written informed consent to participate.

Seventy participants were included in the present study, but eight had to be excluded because the whole cranial vault was not included in the MR images. The excluded participants were proportionally more males. Age, education, and mini-mental state examination (MMSE) scores did not differ between the remaining and the excluded participants. Of the remaining participants 23 were males and 39 females with a mean age of 66 years, a mean education of 11 years and a median MMSE score of 28.5. Twenty-five of the participants were demented, eight had either subjective or mild cognitive impairment, and 29 were normal controls.

The participants and the manual ICV delineations are the same ones as in a previous study by Klasson et al. [4].

### MR acquisition

A coronal MP-RAGE sequence from a 1.5-T Siemens Symphony scanner was used in the present study. The acquisition parameters were: echo time = 2.38 ms, field of view = 250 × 203 mm, flip angle = 15 °, inversion time = 820 ms, matrix size = 512 × 416, acquisition pixel spacing = 1.0 × 1.0, reconstruction pixel spacing = 0.49 × 0.49 mm, repetition time = 1610 ms, slice thickness = 1 mm, bandwidth = 220, number of slices = 192, mean acquisition time = 1.97 min and transmit coil = body.

### Manual estimates using fully delineated ICV

**Fig. 1:**
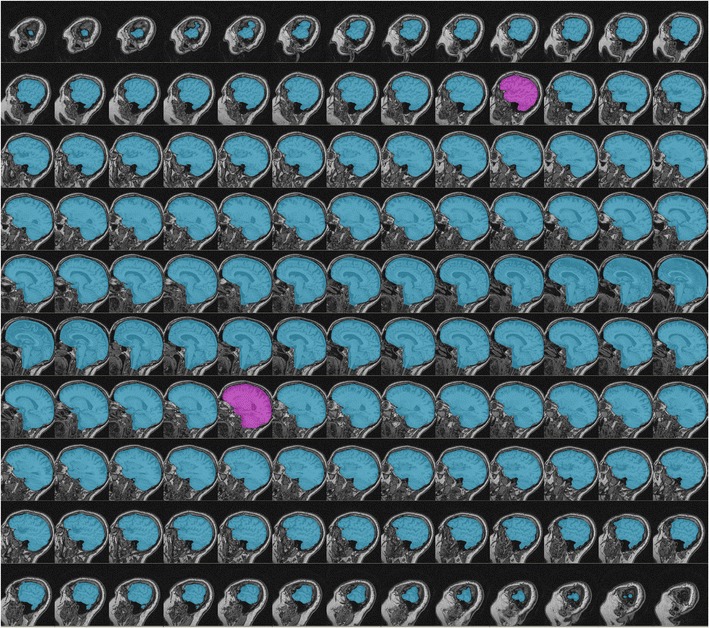
Example of a fully delineated intracranial volume. Sagittal magnetic resonance images from one of the participants. The manually delineated intracranial areas are highlighted in blue and pink. Using method 3 from the present study, only the pink areas would have to be delineated to achieve an estimate with high Pearson correlation to the fully delineated intracranial volume

The MR images were preprocessed, including a down sampling to 1 mm cubic voxels [4], and the whole cranial vault manually delineated in all slices following the dura mater in sagittal orientation. The delineation followed the landmarks described by Eritaia et al. [9]. Figure 1 shows one of the fully delineated ICVs.

To enable ICAs with coronal, sagittal and transversal orientation, the sagittal delineations where reconstructed into binary ICV masks using the MATLAB (version R2012b) function inpolygon. The ICV masks were manually rotated to align to the anterior and posterior commissure (ACPC) axis and so that the longitudinal fissure lay vertically in the transversal and coronal views. The rotations were made using the imwarp function with cubic interpolation.

### Estimation methods

Four ICV estimation methods were validated through comparisons with the fully delineated ICVs. Besides the delineation of ICAs, methods 2 and 3 utilise the perpendicular intracranial width, estimated as the distance between the outermost edges of the cranial vault in the direction perpendicular to the plane of the ICAs. The edges were found automatically by locating the outermost voxels in the ICV masks. A manual approach for one of the methods is suggested in Sect. 4.2.

#### Method 1

Method 1 uses the mid-sagittal ICA, which is determined by the slice where the cerebral aqueduct is most prominent, as an estimate of ICV.

#### Method 2

Method 2 uses a single ICA in a given orientation multiplied by the intracranial width perpendicular to the plane of the ICA.

#### Method 3

**Fig. 2:**
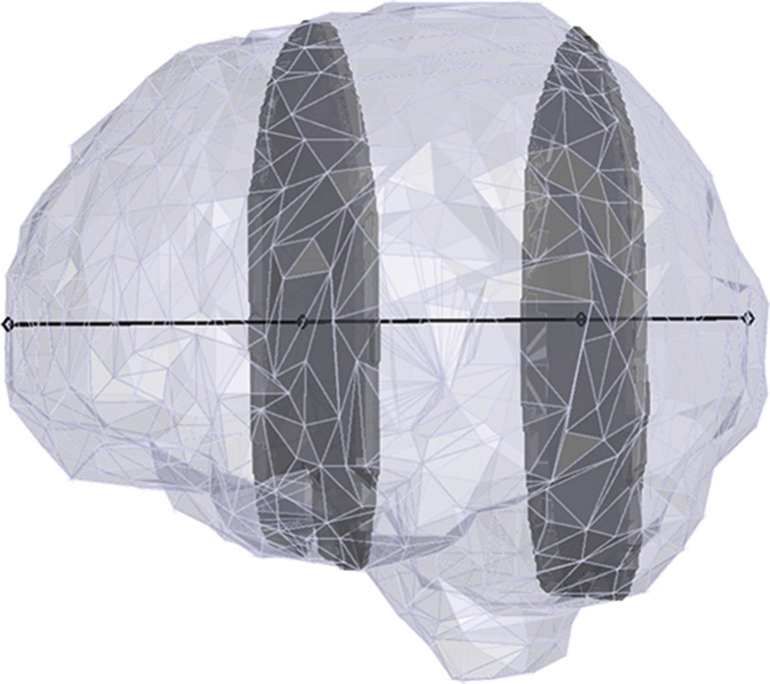
Intracranial volume estimation using method 3 in coronal orientation. The grey surface encloses the intracranial volume (ICV) to be estimated. The black line shows the coronal intracranial width, and the grey areas are two delineated coronal intracranial areas (ICAs). To calculate the ICV estimate using method 3, the coronal intracranial width is multiplied by the sum of the two ICAs

Method 3 uses the sum of two ICAs in a given orientation multiplied by the intracranial width perpendicular to the plane of the ICAs. Figure 2 illustrates the calculation of an estimate using method 3.

#### Method 4

Method 4 uses a shape-preserving piecewise cubic interpolation on two ICAs in a given orientation. To get an interpolation of the whole cranial volume, and not just the volume between the two ICAs, the fact that the ICA is zero mm^2^ beyond the cranial borders is used. The interpolation was performed using the MATLAB (version R2015b) function interp1, and the sum of the resulting areas was used as the ICV estimate. Movie 1, in the supplementary material, illustrates the calculation of an estimate using method 4.

The positions of the ICAs were determined as percentages, below referred to as position indices, of the perpendicular intracranial width. The position indices were calculated starting from the participants’ right side in sagittal orientation, anteriorly in coronal orientation and superiorly in transversal orientation, e.g., a sagittal area with an index of 78 would be located at 78% of the intracranial sagittal width. For a participant with an intracranial sagittal width of 150 mm, the ICA would thus be the one closest to being 117 mm (150 mm *78/100) from the right side of the cranial vault.

### Method evaluation and validation

For method 1, the Pearson correlation with the 62 fully delineated ICVs was calculated. Intra- (N. Klasson) and inter-rater (S. Skau) re-estimations were also performed on all 62 MR acquisitions. The re-estimations were performed at least 6 months after the initial estimations, and both raters were blinded to participant data and to the previous estimations.

For methods 2–4, the 62 ICV masks were randomly separated into a training set and a validation set of equal size. To find the most appropriate ICA position indices for the respective orientation, all possible index combinations using 0.5% intervals were evaluated in the training set. The indices that resulted in estimates with the highest Pearson correlation with the fully delineated ICVs were used to define the methods, which were then validated using the validation set.

During the training step, for each given position index, the Pearson correlation calculated was an average correlation of the estimates from the given ICA and estimates using the two closest ICAs. For methods using two ICAs, the average correlation was calculated for all possible combinations of pairs of ICAs (3^2^ possible combinations for each pair of position indices evaluated). This was done to reduce the risk of finding methods where the use of a neighbouring ICA would give much worse estimates. The resulting methods were then validated in the validation step without considering neighbouring ICAs.

### Further evaluation of method 3

Two additional evaluations were made to determine whether method 3 could be simplified when using sagittal ICAs. These evaluations were made on the validation sample and the effect measured by the resulting estimates’ correlation with the fully delineated ICVs.

#### Starting side

The position indices found for sagittal ICAs start at the patients’ right. As the skull is fairly symmetrical, the indices might be usable from either side. Thus, we determined (1) the effect of calculating the estimates using the indices from the patients’ left and (2) the effect of calculating the estimates using the indices randomly from either the patients’ left or right. The second analysis was done 500 times.

#### The effect of head rotation

To evaluate the effect of transversal and coronal head rotation on the validity of the estimates, we determined: (1) the effect of rotation when all MR acquisitions are equally rotated −10 to 10 degrees in either or both orientations and (2) the effect of rotation when all MR acquisitions are randomly rotated within a given range between −10 to 10 degrees in both orientations. The second analysis was done 500 times.

### Statistical tools

The Pearson correlations were calculated in MATLAB (version R2015b) using the function corrcoef. The correlations found for methods 2 to 4 were then compared using the confidence intervals for differences between overlapping correlations [14].

## Results

**Fig. 3:**
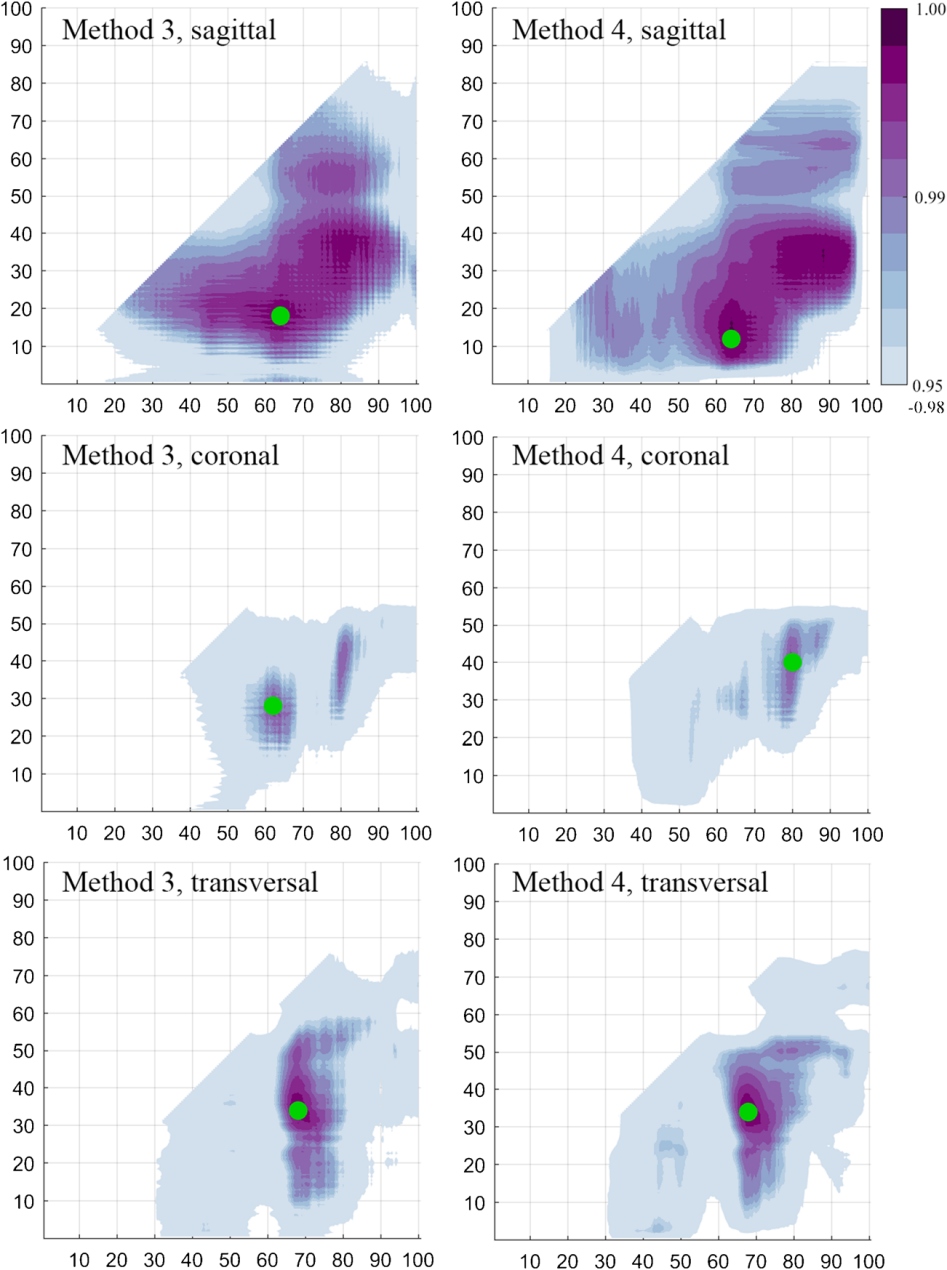
Pearson correlations found during the training step. The y- and x-axes show the position indices (in percent of the intracranial width) while the colours show the correlations found during the training step with the fully delineated intracranial volumes. No colour indicates a correlation less than 0.95 or that the given combination of position indices was not evaluated. The green points are the position indices that later were validated during the validation step

From the training step of methods 3 and 4, Fig. 3 visualises the found average Pearson correlations between fully delineated ICV and estimates using different position indices. Both methods showed the highest correlations around the same combinations of position indices. While all three orientations of the ICAs resulted in estimates with correlations close to one, the use of sagittal ICAs provided a wider range of position indices that resulted in estimates with high correlations. The optimal position indices for method 3, when using sagittal ICAs, were 17.5 and 64.0% of the perpendicular intracranial width and, for method 4, 12.0 and 64.0%.

**Fig. 4:**
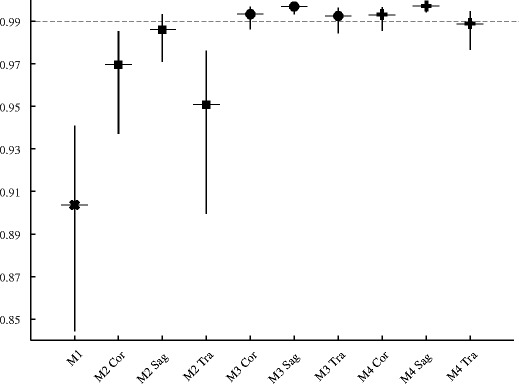
Pearson correlations found during the validation step. The correlations with 95% confidence intervals for method (M) 1 are shown with an x marker, for method 2 with square markers, for method 3 with circle markers and for method 4 with plus sign markers. For each method, except for method 1, the correlations for coronal (Cor), sagittal (Sag) and transversal (Tra) intracranial areas are presented from left to right. The dashed line marks the y-coordinate for correlations of 0.99

Table 1 lists the optimal position indices obtained from the training step (the green dots in Fig. 3) with the corresponding Pearson correlations and percentage errors from the validation step. Table 1 also lists the Pearson correlation with the fully delineated ICV for the estimate from method 1. The highest correlation in the validation step, *r* = 0.997, was reached by method 3 and 4 when using sagittal ICAs. The correlations from the validation step are also visualised in Fig. 4. The smallest average percentage error was achieved by method 4 when using coronal ICAs and the smallest variance in percentage error by method 4 when using sagittal ICAs.

**Table 1:** Results

Method	Orientation	Index 1	Index 2	Correlation (CI)	Absolute percentage error (SD)	Percentage error (SD)
1	Sagittal	–	–	0.904 (0.844–0.941)	–	–
2	Coronal	51.0	–	0.951 (0.899–0.976)	76.2 (5.8)	76.2 (5.8)
2	Sagittal	31.0	–	0.986 (0.971–0.993)	27.0 (2.2)	27.0 (2.2)
2	Transversal	67.0	–	0.970 (0.937–0.985)	63.5 (4.0)	63.5 (4.0)
3	Coronal	27.5	62.0	0.992 (0.984–0.996)	189.5 (3.6)	189.5 (3.6)
3	Sagittal	17.5	64.0	0.997 (0.993–0.998)	125.7 (1.8)	125.7 (1.8)
3	Transversal	33.5	68.0	0.993 (0.986–0.997)	178.7 (3.2)	178.7 (3.2)
3*	Sagittal	17.5	64.0	0.998 (0.995–0.999)	128.5 (1.5)	128.5 (1.5)
3**	Sagittal	17.5	64.0	0.995 (0.001)	127.1 (2.1)	127.1 (2.1)
4	Coronal	40.0	79.5	0.989 (0.977–0.995)	1.4 (1.1)	−1.0 (1.5)
4	Sagittal	12.0	64.0	0.997 (0.994–0.999)	6.7 (0.7)	−6.7 (0.7)
4	Transversal	33.5	68.0	0.993 (0.985–0.997)	5.4 (1.2)	5.4 (1.2)

Indices 1 and 2 are the position indices found during the training step. The correlations with 95% confidence intervals (CI) and the percentage errors with standard deviations (SD) are from the validation step when applying the found position indices, except for method 1 where the estimates were compared with all 62 fully delineated intracranial volumes from the beginning. *Position indices starting from the patients’ left. **Position indices randomly starting from the patients’ left or right, mean correlation and standard deviation from 500 tests

### Starting side

See Table 1. For method 3 with sagittal ICAs, there was a negligible difference in the correlation to fully delineated ICV between using the position indices consistently from the patients’ left and from the right, respectively. When using the indices randomly from either the patients’ left or right, the correlation dropped slightly.

### Comparison of correlations

**Fig. 5:**
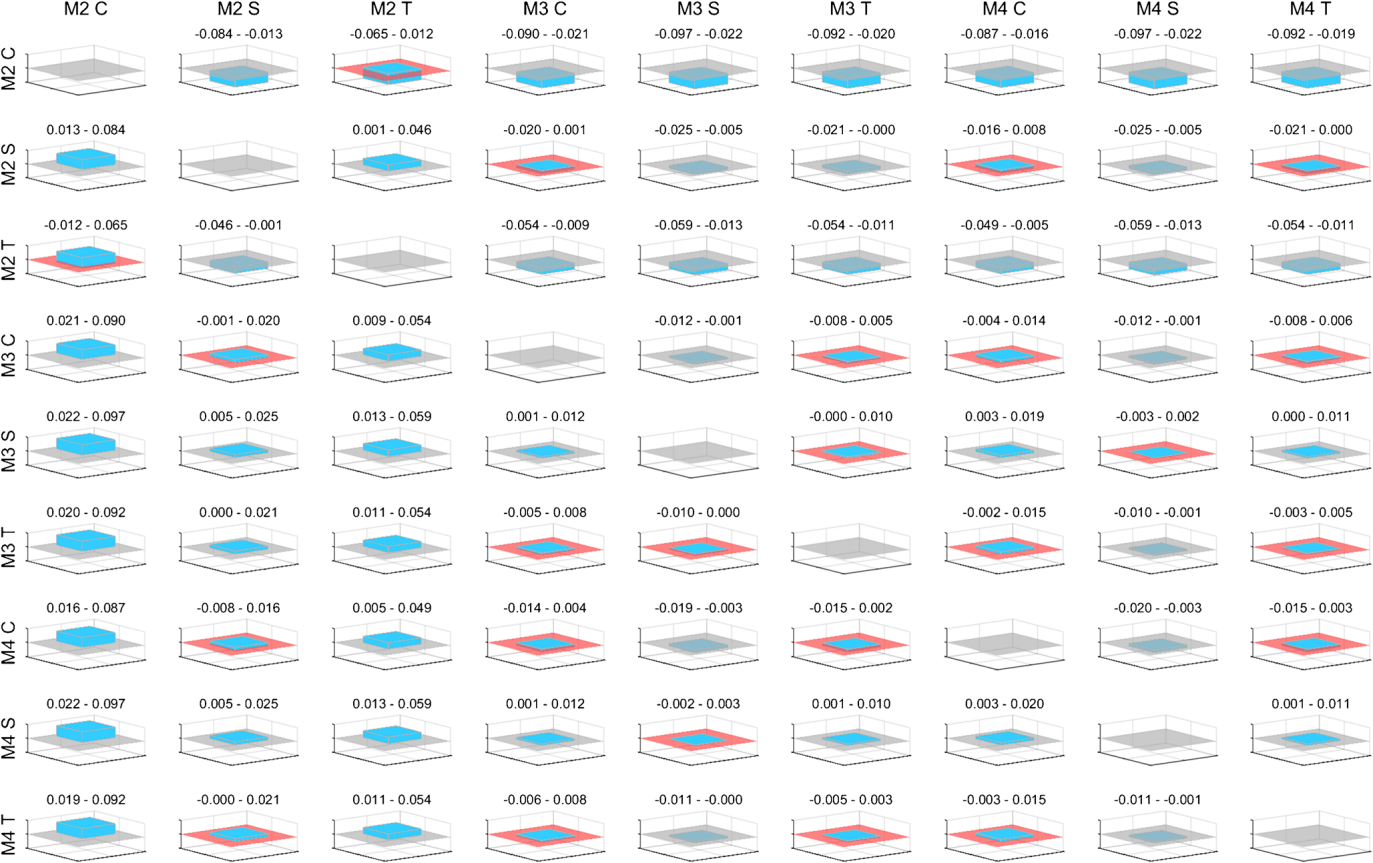
Comparison of correlations. With 95% confidence, the actual differences in correlations between methods 2 to 4 (M2–4) are within the height of the plotted boxes. The unit of the y-axes is the Pearson correlation and ranges between −0.1 and 0.1. Above each plot, the confidence intervals are also given in numbers rounded to the nearest thousandth. The titles of the columns tell which correlation was used as minuend and the titles of the rows which correlation was used as subtrahend when calculating the confidence intervals. The planes denote a correlation difference of zero and are red when the confidence intervals pass through zero, indicating that the actual difference between the two compared methods might be zero. C (coronal), S (sagittal) and T (transversal) denote the orientation of the intracranial areas used

Figure 5 visualises the confidence intervals for the differences in correlations among methods 2, 3 and 4. With 95% confidence, method 4 with sagittal ICAs will result in estimates that have a higher correlation to fully delineated ICVs than all other estimates, except those from method 3 with sagittal ICAs.

### The effect of head rotation

**Fig. 6:**
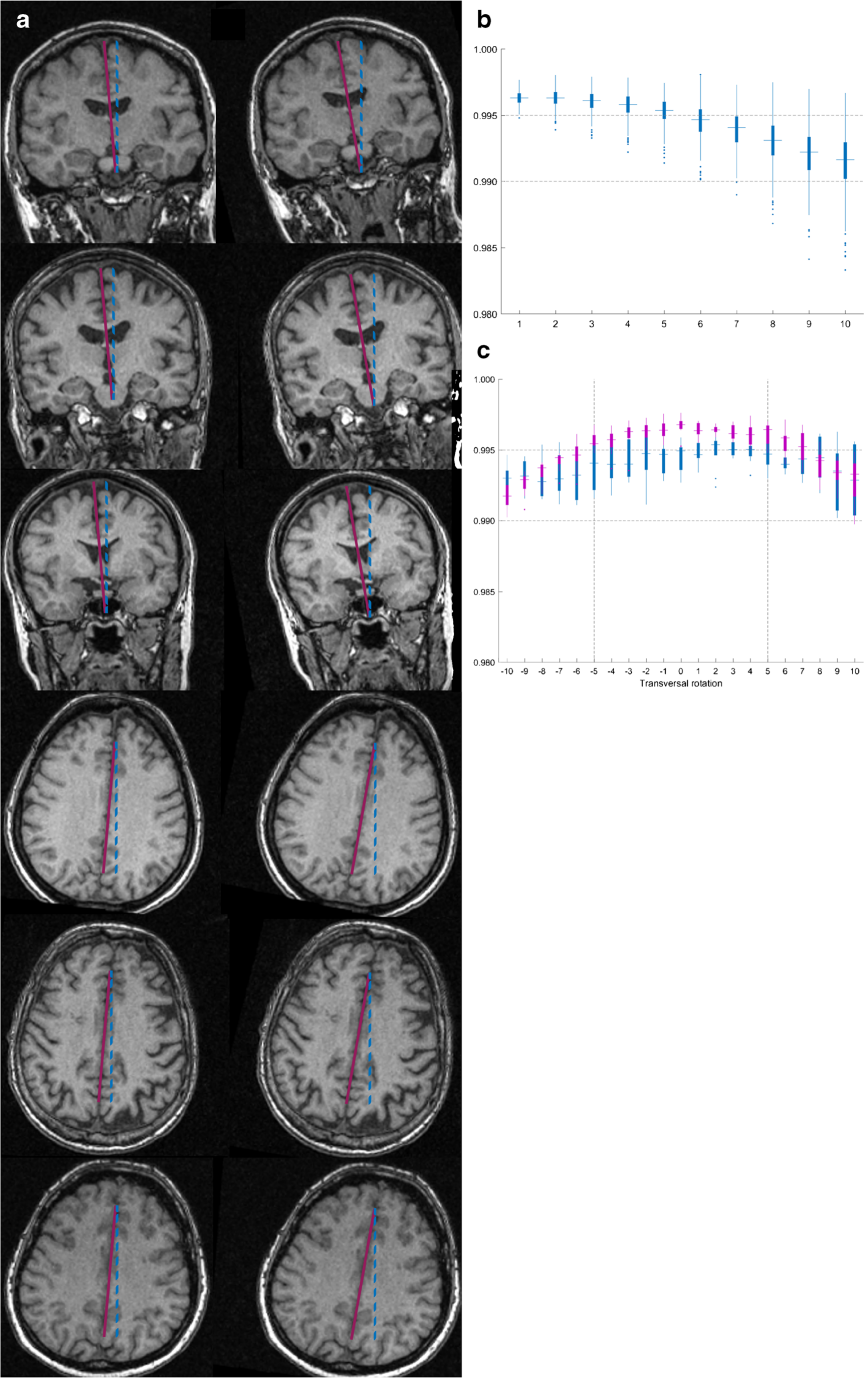
Head rotation when using method 3 in sagittal orientation. **a** Three different magnetic resonance (MR) acquisitions that have been misaligned in coronal and transversal orientation. The left column illustrates a 5-degree rotation (purple line) and the right column a 10-degree rotation (purple line) from the preferred alignments (blue line). **b** The effect of random rotation in coronal and transversal orientation. The maximum degree of rotation is given by the x axis and the effect on the correlation of the estimate with fully delineated intracranial volumes (ICV) by the y axis. **c** The effect of non-random rotation in coronal and transversal orientation between MR acquisitions. The degree of transversal rotation is given by the x axis and the effect on the correlation of the estimate with fully delineated ICV by the y axis. The purple box plots illustrates the distribution of the resulting correlations when the coronal rotation is within 5 degrees and the blue box plots when the coronal rotation is beyond 5 degrees

Figure 6 illustrates how rotation of the head from the preferred alignment will reduce the correlation of the estimate with the fully delineated ICVs when using method 3 with sagittal ICAs. On average, a misalignment in coronal and transversal orientation did not reduce the correlation below 0.995 when within 5 degrees from the preferred alignment and not below 0.99 when within 10 degrees.

### Intra- and inter-rater correlation

The intra- and inter-rater Pearson correlations for the mid-sagittal ICA delineations were 0.997 and 0.995 respectively.

## Discussion

Delineation of two ICAs is enough to achieve adequate estimates of ICV. The sum of two selected ICAs multiplied by the intracranial width perpendicular to the ICAs was shown to have a Pearson correlation with the fully delineated ICV above 0.99. Even when using only one sagittal ICA positioned at 31% of the intracranial sagittal width (from the patient’s right side), a correlation of 0.986 with the fully delineated ICV was achieved.

Shape-preserving piecewise cubic interpolation, instead of the sum of two ICAs multiplied by the intracranial width, barely improved the Pearson correlation with the fully delineated ICV. However, with the cubic interpolation, the percentage error of the estimates was smaller. Cubic interpolation with coronal ICAs resulted in the smallest absolute percentage errors (1.4 ± 1.1%), while using sagittal ICAs resulted in the smallest variance of the percentage errors (6.7 ± 0.7%). When using linear regression, where the mean percentage error is of less importance, sagittal ICAs are still preferable over coronal ICAs (Fig. 5).

The Pearson correlation between the mid-sagittal ICA and the fully delineated ICV is similar to those shown in two previous studies (*r* = 0.88–0.89) [10, 11], but smaller than the Spearman rho found for a child population (rho = 0.96) [15]. In the study by Nandigam et al., where 6.5-mm slices with 1.5-mm slice gaps were used, the average of the two most mid-sagittal ICAs resulted in estimates with a Pearson correlation of 0.94 [10]. Delineation of two to three ICAs with 50-mm spacing may result in correlations around 0.96–0.99 [4] and if using four specific ICAs with three different orientations in a correlation of 0.99 [16]. Previously, it has been necessary to delineate multiple ICAs with linear spacing less than 35 mm to achieve correlations above 0.99 [4, 9]. The best available automatic methods tend to show correlations to manual or semi-manual ICV estimates of around 0.97–0.99 [6, 8, 17].

### Head position

When delineating one or two ICAs, the position of the head in the images becomes more important than when using multiple ICAs. In the present study, all heads were uniformly aligned in all three planes. However, depending on the orientation of the ICAs to be segmented, the planes that need aligning differ, e.g., the alignment of the head to the ACPC axis is unnecessary when delineating sagittal ICAs. The effect of head rotation on method 3 was evaluated for sagittal ICAs. By this evaluation, it was seen that coronal and transversal misalignments within 5 degrees from the preferred alignment in general could be expected to reduce the correlation with fully delineated ICVs to around 0.995, and never below 0.99.

### Suggested method

To achieve good estimates of ICV for variance reduction when using linear regression, we suggest delineation of two sagittal ICAs multiplied by the intracranial sagittal width. This means that the following steps should be followed: (1) if the head is tilted more than 5 degrees in the coronal or transversal view, rotate the MR images so that the longitudinal fissure lies vertically in these views; (2) count all sagittal slices within the cranial vault; (3) multiply the slice count with 0.175 and 0.64 to get the positions of the ICAs to delineate; (4) find the sagittal slices that are closest to these two positions (counted from either of the two outermost sagittal slices); (5) delineate the ICAs at the given slices; (6) sum the two ICAs and multiply by the slice count.

### Limitations

The correlations found for the different methods in the present study do not include rater variance. Depending on the rater, the validity of the methods might be lower than established.

The generalisability of the results to other populations and other MR sequences cannot be determined from the present study alone. In the present study, the MR sequence was chosen out of convenience, as fully delineated ICVs were already available from a previous study [4]. The use of T2-weighted images might improve the reliability of the estimates because of better contrast between the skull and cerebrospinal fluid.

As the evaluated methods are based on the delineation of the dura mater, the validity of the estimates should not vary with brain atrophy. However, this assumption was not tested.

## Conclusion

A simple and adequate ICV estimate for use in linear regression can be achieved by delineating two sagittal ICAs at 17.5 and 64% of the intracranial sagittal width. The Pearson correlation with fully delineated ICV was shown to be 0.997. The estimate takes no more than a few minutes per ICV to acquire and correlates more strongly with fully delineated ICV than estimates from any established automatic method.

## Electronic supplementary material

Movie 1 Intracranial volume estimation using method 4. The grey surface encloses the intracranial volume (ICV) to be estimated. The black line that is then drawn gives the intracranial coronal width. By multiplying the intracranial coronal width by two position indices, the position of the intracranial areas (ICA) to delineate is determined. The grey areas represent the delineations of the ICAs at the given positions. Knowing that the ICA is zero mm^2^ beyond the cranial borders, and knowing the delineated ICAs and their positions, the ICV can be estimated by a shape-preserving piecewise cubic interpolation. The sum of the resulting areas is the ICV estimate. (AVI 45234 kb)

## Abbreviations

ICV: Intracranial volume
MR: Magnetic resonance
ICA: Intracranial area
MMSE: Mini-mental state examination
MCI: Mild cognitive impairment
CI: Confidence interval
SD: Standard deviation
ACPC: Anterior and posterior commissure
r: Pearson correlation coefficient
M: Method
Tra: Transversal
Sag: Sagittal
Cor: Coronal

## Footnotes

1 Knowing that actual intracranial volume (ICV) explains 16% of the variance of hippocampal volume, an ICV estimate has to explain (1–0.01/0.16)*100 = 93.75% of that variance to reduce it to 1 %. As long as the error in the ICV estimate is random and independent of hippocampal volume, a Pearson correlation of 0.9375^0.5^ to actual ICV is needed.
